# Exploring the interplay of karyotype, hormones, sexuality, and body image perception in individuals with Turner syndrome

**DOI:** 10.1007/s40618-024-02521-0

**Published:** 2025-02-13

**Authors:** Chiara Tarantino, Ludovica Vincenzi, Francesco Angelini, Alessandra Tomaselli, Francesco Carlomagno, Elena Rosato, Riccardo Pofi, Andrea Lenzi, Carlotta Pozza, Marianna Minnetti, Matteo Spaziani, Andrea M. Isidori, Emilia Sbardella

**Affiliations:** 1 Section of Medical Pathophysiology and Endocrinology, Department of Experimental Medicine, Sapienza University of Rome, Rome, 00161 Italy; 2 Endocrine and Andrological Regional Rare Disease Center (Endo-ERN accredited), Policlinico Umberto I, Rome, 00161 Italy; 3 Oxford Centre for Diabetes, Endocrinology and Metabolism, NIHR Oxford Biomedical Research Centre, Churchill Hospital, University of Oxford, Oxford, UK; 4 Department of Therorethical and Applied Sciences, eCampus University, Novedrate CO, Italy

**Keywords:** Female sexual dysfunction, Sexuality, Rare disease, Turner Syndrome, Androgen deficiency, 45,X

## Abstract

**Purpose:**

Most patients with Turner Syndrome (TS) require Hormone Replacement Therapy (HRT). Androgen levels could be compromised due to both ovarian insufficiency and HRT. Despite this, the association between androgen deficiency, sexual health, and body image perception remains underexplored in these patients. This study aimed to assess hormone levels, sexual function, and body image perception in women with TS, categorized by karyotype and HRT regimen.

**Methods:**

A cross-sectional analysis of 29 patients with TS was performed. Clinical, hormonal, and ultrasonographic pelvic parameters were evaluated. Sexual function and body image perception were measured using the Female Sexual Function Index (FSFI) and the Body Uneasiness Test (BUT) questionnaires.

**Results:**

The cohort included individuals with X chromosome monosomy (Group A), structural X chromosome alterations in some cell lines (Group B) or in all cell lines (Group C), and cells with 46, XX karyotype and monosomy (Group D). Group A and B compared to Group D displayed lower calculated free testosterone (*p* = 0.006, *p* = 0.032) and free androgen index levels (*p* = 0.007, *p* = 0.025). DHEA-S values differed between groups A and D (*p* = 0.043) and between groups A and C (*p* = 0.044). Sexual activity was reported by approximately half of patients (51.7%), with 57% of them presenting sexual dysfunction. Additionally, 44.8% exhibited possible body image disorder.

**Conclusions:**

This study acknowledges significant phenotypic differences linked to karyotype in women with TS, highlighting the prevalence of sexual dysfunction and body image dissatisfaction. These findings emphasize the importance of addressing sexual health and body image issues in patients with rare diseases, often neglected in clinical practice.

## Introduction

Turner syndrome (TS) is a rare genetic condition affecting approximately one of 2.500 newborn girls, characterised by hypergonadotropic hypogonadism, short stature, infertility, endocrine and metabolic disorders, and increased risk of cardiovascular and autoimmune diseases [1–3].

The syndrome results from a complete or partial deletion of one X chromosome, with variability in karyotypes including a complete absence of one X chromosome (45,X), chromosomal mosaicism (e.g. 45,X/46,XX; 45,X/47,XXX), the presence of an isochromosome of either the p or q arm, ring chromosomes, or the existence of Y-chromosomal material [2]. This heterogeneity strongly influences phenotype: 45,X monosomy presents with an overt clinical picture, while mosaicism (e.g. 45,X/46,XX) predicts a less prominent clinical presentation, ensuring the ability to experience pubertal development and spontaneous menarche [4, 5].

Ovarian insufficiency in TS begins early in foetal life, leading to primordial follicles depletion and the formation of “streak gonads” resulting in hypergonadotropic hypogonadism [6]. Most patients with a 45,X karyotype experience early ovarian failure, preventing complete pubertal development [6–8]. About 30% of girls with TS undergo some pubertal development and 10–20% achieve spontaneous menarche, though spontaneous pubarche occurs in most patients regardless of the karyotype [9–12]. Therefore, most of these patients require hormone replacement therapy (HRT) to develop secondary sexual characteristics and maintain psychophysical well-being in adulthood, including sexual and bone health [1, 13].

TS can be diagnosed at different ages, depending on the chromosomal alteration, with early diagnosis being critical for optimal management and potential fertility preservation [14–16].

Despite limited data, ovarian insufficiency could also reduce androgen levels [17]. Additionally, HRT with oestrogens can negatively influence androgen levels, further reducing the quantity of bioavailable androgens, with possible negative consequences on the sexual health of these patients [18, 19]. In this regard, over the past twenty years, the role of androgens in women’s sexual health has gained increasing attention [20–23].

Genetic studies suggest that many aspects of human sexuality, including behaviours like mating strategies, sexual orientation, and female sexual dysfunction (FSD), may have a genetic basis [24]. This is particularly relevant in TS, where chromosomal anomalies may influence the relationship between hormonal profiles, sexual health, and body image perception.

Among patients with TS, despite the potential impairment also due to reduced androgen levels, sexual health is widely unexplored. Some studies have reported that patients with TS are less likely to be sexually active than the general population or healthy control. However, interestingly, these studies have also reported no overall compromise in sexual function [25–27]. Additionally, it appears that patients with TS have a prevalent dissatisfaction with their body image, which may also affect sexuality to some extent [28]. Therefore, the present study aims to assess and integrate aspects related to circulating androgen concentrations, sexual function, and body image perception in patients with TS, being the first to categorise them according to different karyotypes.

## Materials and methods

### Participants

The present cross-sectional study was conducted at the Endo-ERN and Regional Rare Endocrine and Andrological Disease Centre at the Department of Experimental Medicine of Sapienza University of Rome, Italy (“Policlinico Umberto I” Hospital), between December 2023 and March 2024. Investigations were carried out to assess clinical and hormonal parameters, sexual function, and body perception in 30 patients diagnosed with TS. Inclusion criteria were as follows: age between 18 and 50 years, diagnosis of TS confirmed by a karyotype analysis of postnatal peripheral blood, conducted on at least 40 metaphases. Exclusion criteria included: prepubertal patients, patients undergoing HRT for less than one year at the time of evaluation, patients with any overt or uncontrolled psychiatric disorders, patients using psychotropic medications (e.g., SSRIs, antipsychotics) or any medications known to affect sexual function. The study was performed in accordance with the principles of the Declaration of Helsinki, and patient enrolment was authorised by the Local Ethics Committee (Approval ref. CE 4945, Protocol no. 0897/23 of November 13, 2023). The results were recorded under an anonymous alphanumeric code for each patient.

### Clinical evaluation

For all patients, a comprehensive clinical evaluation encompassing demographic and clinical parameters was performed. Anthropometric assessments were conducted using a Harpenden stadiometer to measure height and calibrated scales to determine weight. Waist and hip circumferences were assessed using a measuring tape, with precision to the nearest 0.1 cm. From these measurements, we calculated the body mass index (BMI) and Waist-to-Hip Ratio (WHR) for each patient. Additionally, we investigated the participants’ medical backgrounds, focusing on discerning the presence or absence of spontaneous menarche, hearing impairment, Hashimoto’s thyroiditis, and prior treatment with recombinant human growth hormone (rhGH). We also evaluated the potential influence of comorbidities such as hypertension, dyslipidemia, and glucose intolerance or type 2 diabetes mellitus, along with their respective treatments.

### Hormonal evaluation

Hormonal tests were conducted at the Laboratory of the Department of Experimental Medicine, Policlinico Umberto I (Rome), using various established methodologies. Serum levels of thyroid-stimulating hormone (TSH), free triiodothyronine (fT3), free thyroxine (fT4), luteinizing hormone (LH), follicle-stimulating hormone (FSH), oestradiol (E2), sex hormone-binding globulin (SHBG), dehydroepiandrosterone sulfate (DHEA-S), total testosterone (TT), and prolactin (PRL) were quantified using chemiluminescence methodology (CMIA, Architect System by Abbott Laboratories, IL, USA), with limits of detection (LOD) of 0.0025 µIU/mL, 1.0 pg/mL, 0.4 ng/dL, 0.07 mIU/mL, 0.05 mIU/mL, 10 pg/mL, 0.1 nmol/L, 0.25 ng/mL, 0.28 nmol/L, and 0.6 ng/mL respectively. ∆4 was measured with radioimmunoassay (RIA; Cisbio Bioassays, Codolet, France) with LOD 5 ng/dL, whereas the intra- and inter-assay CVs were 7.4% and 8.1%, respectively, at 168 ng/dL. Cortisol was measured by RIA (Beckman Coulter): the LOD was 5 nmol/L, and the intra- and inter- assay CVs were 4.9% and 7.8%, respectively, at 447.8 nmol/L. Calculated free testosterone (cfT) and free androgen index (FAI) were obtained using the Vermeulen’s formula, assuming a fixed albumin concentration of 4.3 g/dL, and the ratio of TT to SHBG levels, expressed in nmol/L, and then multiplying by 100, respectively.

### Questionnaires

Sexual function and subjective perception of body image were investigated through the administration of anonymous questionnaires, the FSFI (Female Sexual Function Index) and the Body Uneasiness Test (BUT) A and B. Both questionnaires were administered confidentially and although the questionnaire was self-reported, a clinician was available for any clarifications and to ensure all items were answered.

The BUT (A and B) is a self-assessment scale which evaluates body uneasiness and dissatisfaction [29]. The BUT-A includes 34 items, encompassing 5 distinct subscales: weight phobia, body image concerns, avoidance, compulsive self-monitoring, and depersonalization; BUT-B consists of 37 questions assessing dissatisfaction with body parts and function [29]. Responses are evaluated on a 6-point Likert-type scale, ranging from “never” to “always” with higher scores indicating greater impairment. The overall average score BUT-A is used to measure the severity of body uneasiness through the Global Severity Index (GSI) [29]. A GSI score exceeding 1.2 reports a potential susceptibility to discomfort concerning one’s body. BUT-B scores can be aggregated into two global measures: Positive Symptom Total (PST) and Positive Symptom Distress Index (PSDI) [29].

Participants were systematically evaluated using the FSFI, one of the most widely used tools for screening FSD. The FSFI, a 19-item self-questionnaire, investigates various aspects of sexuality, including desire, arousal, orgasm, satisfaction, and pain [30]. Each question is scored on a scale from 0 to 1 to 5, and domain scores are multiplied by a specific factor and summed. Higher scores denote a better sexual function and women with an overall score < 26.55 are categorised as at risk for FSD.

### Ultrasonographic evaluations

The evaluation of uterine volume was performed using a 2–5 MHz convex probe for transabdominal ultrasonography, while a 5-7.5 MHz probe was used for the transvaginal approach (Philips iU22). Uterine volume (mL) was calculated using the formula: π/6 × longitudinal diameter (cm) × anteroposterior diameter (cm) × transverse diameter (cm) [31].

### Statistical analyses

Statistical analyses were performed using the software “Statistical Package for the Social Sciences software IBM SPSS Statistics, Version 27” and GraphPad Prism version Version 10.1.1 (GraphPad Software, LLC). Continuous variables were expressed using means, standard deviation (SD), and/or 95% confidence interval for parametric distribution, while median and interquartile range (IQR) at the 25th-75th percentile were utilised for non-parametric distribution. Nominal variables were reported as absolute frequencies and percentages. Categorical variables were compared using the Chi-square test or Fisher’s test, with Bonferroni correction applied for multiple comparisons. The groups were compared using independent samples Student t-tests for unequal variances and using Mann–Whitney U for normally distributed continuous variables between two independent groups. For comparison among multiple groups, ANOVA was applied when variables followed a normal distribution. For non-normal distributions, the Kruskal-Wallis test was used. The Benjamini-Hochberg procedure was applied with a false discovery rate set at 5% to account for multiple comparisons. Additionally, ANCOVA was used to assess the effect of karyotype (predictive variable) on different outcome variables, adjusting for the type of therapy (covariate).

In all analyses, a value of *p* < 0.05 was considered statistically significant.

## Results

Among the 35 patients initially screened for study inclusion, 6 were found to be ineligible based on the exclusion criteria. The remaining 29 patients with TS were enrolled. Among these, 7 presented an X chromosome monosomy (**Group A**, 24%), 15 exhibited structural alterations of the X chromosome, further divided into two groups based on the presence of X chromosome monosomy in some cell lines (**Group B**, 38%) or in all cell lines (**Group C**, 14%). Furthermore, 7 patients had a variable percentage of cells with 46, XX karyotype associated with monosomy (**Group D**, 24%). The classification of karyotypes is detailed in Table 1. Two patients belonging to Group B underwent prophylactic gonadectomy after detection of the Y chromosome.

**Table 1:** Subdivision into subgroups based on karyotype and their respective percentage frequencies

Group	Karyotype	*n*. of patients (%)
**A**	Monosomy 45, X	7 (24)
**B**	Mosaicism with variant	11 (38)
	45, X/46, X, i (Xq)	
	45, X/46, X, r (X)	
	45, X/46, X t (Y; Y)	
	45, X/46, X idic (Yq)	
	45, X/46, X idic (Xq)	
**C**	Variant	4 (14)
	46, X, i (Xq)	
	46, X, idic (Xp)	
**D**	Mosaicism with XX cells	7 (24)
	45, X/46, XX	

The overall mean age at evaluation was 31.4 ± 8.9 years and was comparable across the four groups (*p* = 0.581). The median age at diagnosis of TS was 13 years [IQR 6.0, 19.5]. In Table 2, the different ages at diagnosis are reported.

**Table 2:** Age at diagnosis of TS in years. Data are expressed as median and Interquartile Range in square brackets

	Group A	Group B	Group C	Group D	Total	*p*
**Age at diagnosis** *(years)*	4.0 [0.0–13.0]	11.0 [6.0-15.2]	17.5 [11.2–26.7]	30.0 [7.0–36.0]	13.0 [6.0-19.5]	0.087

Among the patients in the study, 3 were not under HRT. Two of these had spontaneous menarche, regular menstrual cycles and had been pregnant at least once, both belonging to group D. The third patient, from group C, had previously experienced spontaneous cycles, which are now irregular after the autonomous discontinuation of HRT. The remaining 26 patients were on HRT, with 12 patients (46% of the sample) using a transdermal (TD) formulation and 14 patients (54%) on an oral oestrogen-progestin regimen (OS). Further clinical details are available in Table 3.

**Table 3:** Clinical characteristics of patients with TS

Clinical characteristics	
Age, years (mean ± SD)	31.4 ± 8.9
BMI, kg/m2 (mean ± SD)	25.5 ± 5.2
Height, cm (mean ± SD)	152.9 ± 8.6
WC (cm)	80.6 ± 13.8
HC (cm)	92.3 ± 13.6
WHR	0.87 ± 0.1
Age at diagnosis (median [IQR])	13.0 [6.0-19.5]
Uterine volume (median [IQR])	26.7 [21.8–34.8]
Spontaneous menarche	9/29 pt
Hearing impairment	12/29 pt
Hashimoto’s thyroiditis	15/29 pt
Previous therapy with rhGH	13/29 pt
HRT	26/29 pt
HRT TD	12/29 pt
HRT per OS	14/29 pt

Abbreviations: TS Turner Syndrome, BMI body mass index, WC waist circumference, HC hip circumference, WHR waist to hip ratio, IQR: interquartile range, rhGH recombinant human growth hormone, HRT hormone replacement therapy, OS oral administration, TD Transdermal, TS: Turner syndrome. Values are expressed as means ± SD or as median and IQR in square brackets

Spontaneous menarche was observed in 9 patients (31% of the entire sample). Most of these cases occurred in groups C (*n* = 3) and D (*n* = 5), with no instances in group A. Consequently, the distribution of spontaneous menarche was not uniform across the four groups (*p* = 0.001). Specifically, Group A differed significantly from Group D (*p* = 0.042). The remaining 20 patients (69%) required puberty induction therapy. The median age at which patients with spontaneous menarche started HRT was 20 years [IQR 15.7, 32.2].

### Pelvic ultrasound features

**Fig. 1:**
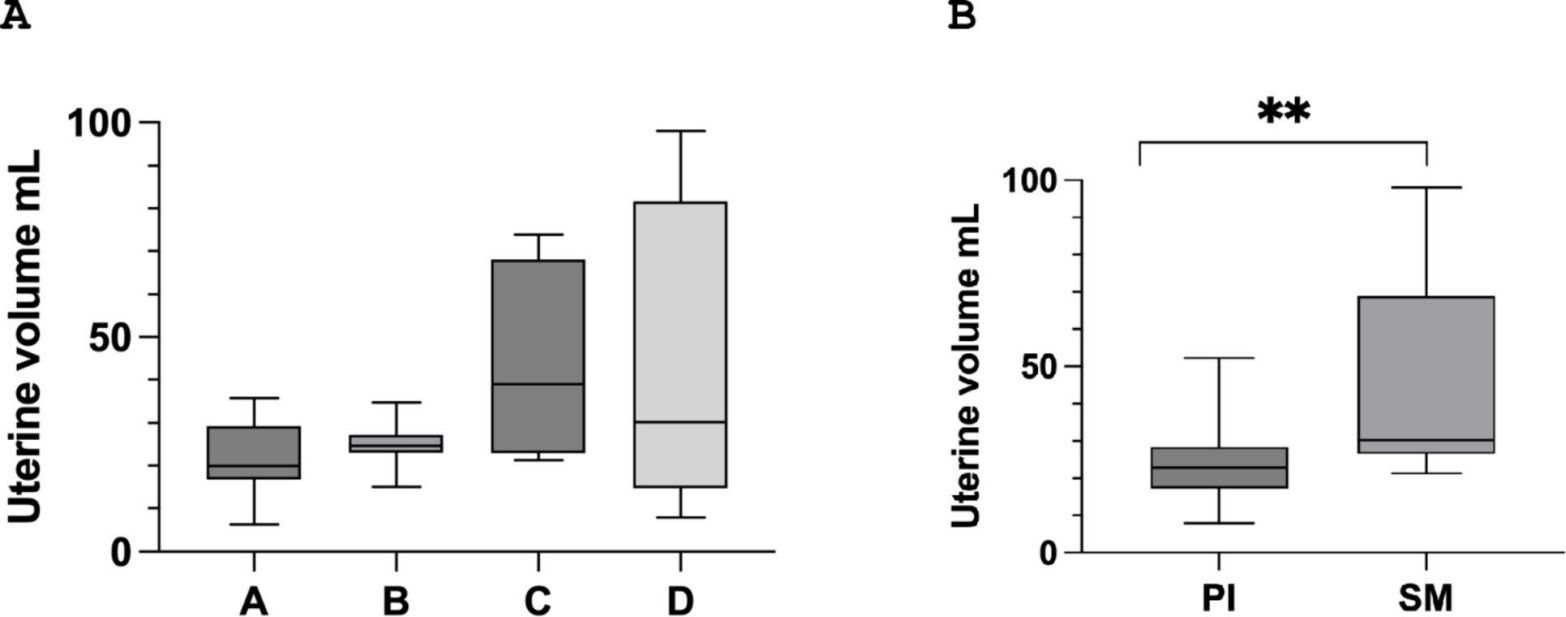
In **A**, the distribution of uterine volume values across the 4 groups is presented. **B** depicts a comparison of uterine volume values between patients with a history of spontaneous menarche (SM) and those with puberty induction (PI). ** *p* < 0.01

The uterine volume distribution was consistent across the four groups, with a median volume of 26.7 mL [IQR 21.8, 34.8] **(**Fig. 1A**)**. Patients who experienced spontaneous menarche showed significantly greater uterine volume (40.05 mL [IQR 27.10, 75.24]) compared to those who underwent puberty induction therapy (23.92 mL [IQR 16.90, 28.45], *p* = 0.004) **(**Fig. 1B**)**.

The ovaries were visualised by ultrasound examination in 65.5% of the participants, although intergroup differences were observed, they did not reach statistical significance (*p* = 0.055). In Group A, the ovaries were visualized in 2 out of 7 patients (28.6%), in Group B in 7 out of 11 patients (63.6%), in Group C in all 4 patients (100%), and in Group D in 6 out of 7 patients (85.7%). However, the differences between individual group comparisons were not statistically significant. Additionally, uterine volume did not differ when the sample was stratified based on the route of HRT administration (*p* = 0.38) or previous treatment with rhGH (*p* = 0.36).

### Hormonal parameters

**Fig. 2:**
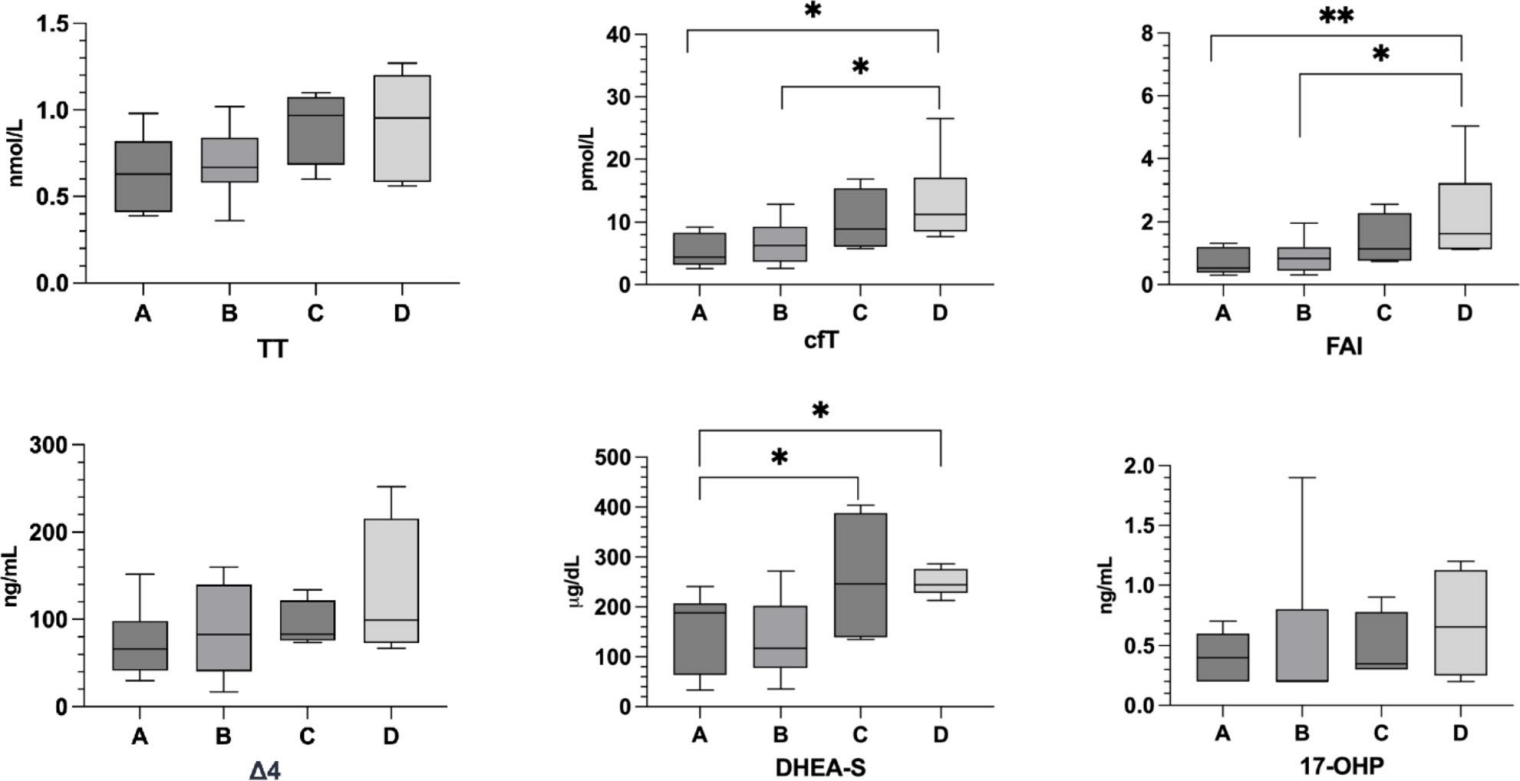
Representation of androgens distributions in patients based on karyotype. * *p* < 0.05, ** *p* < 0.01

Hormonal parameters, distributed among each group, are reported in Table 4. Significant differences were observed in cfT, FAI, and DHEA-S values between participants divided according to the karyotype. Specifically, Group A and B displayed lower cfT levels compared to Group D (*p* = 0.006, *p* = 0.032) and Group A also lower FAI (*p* = 0.007, *p* = 0.025). DHEA-S values were different in the comparison between groups A and D (*p* = 0.043) and between groups A and C (*p* = 0.044) (Fig. 2).

**Table 4:** Hormonal parameters in patients with TS grouped by karyotype

	Group A	Group B	Group C	Group D	*p*
TSH (µUI/mL)	2.8 [1.9–3.8]	2.0 [1.6–2.7]	3.0 [1.7–3.5]	1.3 [1.1–2.4]	0.158
FT3 (pg/mL)	2.8 [2.6-3.0]	2.9 [2.5–3.2]	2.7 [2.4-3.0]	2.8 [2.5–3.3]	0.879
FT4 (ng/dL)	1.2 [1.1–1.3]	1.0 [0.9–1.2]	1.2 [1.0-1.4]	0.9 [0.9–1.2]	0.087
LH (mUI/mL)	6.5 [3.1–11.9]	9.9 [3.0-13.7]	8.0 [0.4–30.7]	4.5 [3.7–6.1]	0.782
FSH (mUI/mL)	16.6 [6.9–33.7]	18.3 [5.0-49.6]	27.5 [3.4–73.6]	11.2 [2.6–23.4]	0.710
E2 (pg/mL)	47.0 [20.0-126.0]	56.0 [59.5–83.0]	60.5 [22.2-115.2]	33.0 [16.0–83.0]	0.902
PRL (ng/mL)	10.1 [9.5–14.3]	10.3 [7.1–16.9]	13.5 [9.3–30.5]	12.2 [5.0–17.0]	0.820
SHBG (nmol/L)	110.1 (66.5-153.7)	86.6 (62.6-110.6)	76.5 (30.3-122.8)	54.2 (23.1–85.3)	0.083
TT (nmol/L)	0.65 (0.45–0.84)	0.69 (0.55–0.83)	0.91 (0.56–1.25)	0.92 (0.60–1.23)	0.094
**cfT (pmol/L)**	**4.40 [3.18–8.30]**	**6.08 [3.67–9.31]**	**8.92 [6.10-15.32]**	**11.26 [8.5–17.0]** ^*,**^	**0.033**
**FAI**	**0.53 [0.39–1.20]**	**0.72 [0.43–1.13]**	**1.14 [0.76–2.28]**	**1.62 [1.12–3.23]** ^*,**^	**0.037**
**DHEA-S (µg/dL)**	**144.4 (67.1-221.7)**	**140.3 (82.16–198.4)**	**257.9 (43.2-472.7)** ^*^	**250.7 (216.7-284.7)** ^*^	**0.036**
Δ4 (ng/dL)	65.8 [41.4–98.1]	83.0 [40.6–140.0]	83.1 [75.8-121.5]	99.3 [73.1-215.75]	0.477
17-OHP (ng/mL)	0.40 [0.2–0.6]	0.20 [0.20–0.80]	0.35 [0.3–0.7]	0.65 [0.25–1.12]	0.615
Cortisol (nmol/L)	527.3 (363.0-691.7)	553.8 (455.2-652.4)	548.0 (306.6-789.4)	388.8 (238.7-538.9)	0.168

Values are expressed as means and 95% confidence intervals in parentheses, or as median and Interquartile Range in square brackets. *p < 0.05 vs. group A, **p < 0.05 vs. group B

No differences were noted in the distribution of TD or OS therapy across the groups. However, within the group that experienced spontaneous menarche, TT (*p* = 0.014), cfT (*p* = 0.019), FAI (*p* = 0.034), and DHEA-S (*p* = 0.008) levels were significantly higher.

**Fig. 3:**
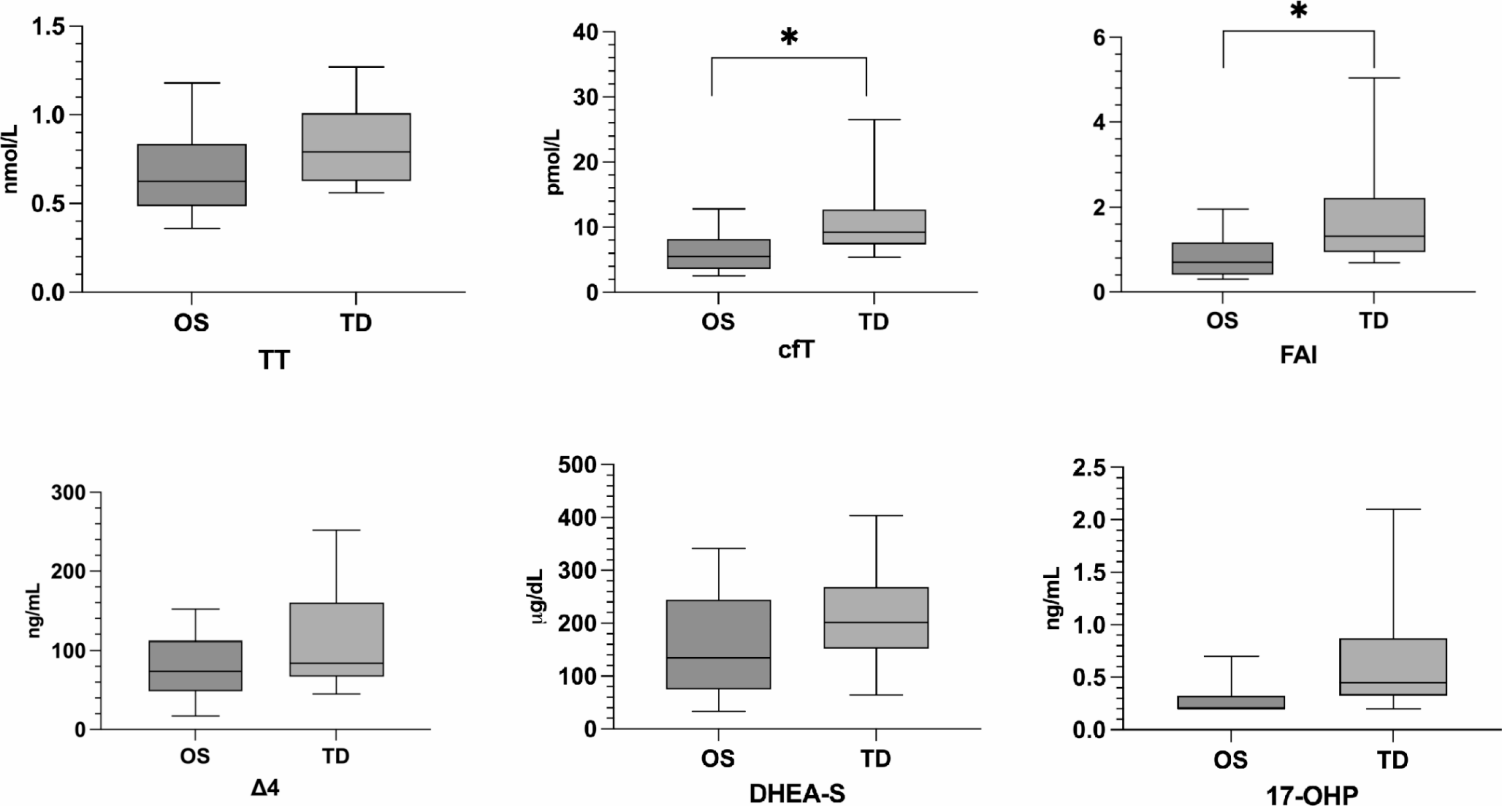
Representation of androgens distributions in patients based on the type of therapy administered. * *p* < 0.05

Significant differences were observed in LH (*p* = 0.020), SHBG (*p* = 0.021), cfT (*p* = 0.014), and FAI (*p* = 0.032) levels, categorising the sample by the route of HRT administration. Notably, the TD therapy group exhibited higher LH, cfT, and FAI levels **(**Fig. 3**)**.

Conversely, SHBG levels were lower in the TD therapy group (*p* = 0.021). The main determinants of SHBG levels included karyotype (*p* = 0.039), route of therapy administration (*p* = 0.046), and age at evaluation (*p* = 0.019) **(**Table 5**)**. Karyotype and route of administration were independently predictive of cfT (*p* = 0.004, *p* = 0.008) and FAI levels (*p* = 0.004, *p* = 0.011) **(**Tables 6 and 7**)**.

**Table 5:** Multiple linear regression of predictor variables for SHBG levels

SHBG	B	*p*	95% confdence interval Lower Upper	
Constant	114.872	0.003	44.906	184.837
**A-D groups**	**-14.076**	**0.039**	**-27.361**	**-0.791**
**OS/TD therapy**	**-33.641**	**0.019**	**-61.151**	**-6.131**
**Age at evaluation**	**1.617**	**0.047**	**0.024**	**3.211**

Abbreviations: B unstandardized coefficient; OS: oral administration; TD: transdermal. Values with *p* < 0.05 are highlighted in bold

**Table 6:** Multiple linear regression of predictor variables for cfT levels

cFT	B	*p*	95% confdence interval Lower Upper	
Constant	0.251	0.053	-1.065	-0.464
**A-D groups**	**0.115**	**0.003**	**0.042**	**0.188**
**OS/TD therapy**	**0.241**	**0.004**	**0.088**	**0.394**

Abbreviations: B unstandardized coefficient; OS: oral administration; TD: transdermal. Values with *p* < 0.05 are highlighted in bold

**Table 7:** Multiple linear regression of predictor variables for FAI levels

FAI	B	*p*	95% confdence interval Lower Upper	
Constant	-0.764	< 0.001	-1.065	-0.464
**A-D groups**	**0.296**	**0.003**	**0.116**	**0.476**
**OS/TD therapy**	**0.145**	**0.002**	**0.059**	**0.231**

Abbreviations: B unstandardized coefficient; OS: oral administration; TD: transdermal. Values with *p* < 0.05 are highlighted in bold

### Sexual function

Approximately half of participants (51.7%) were sexually active, with the likelihood of engaging in sexual intercourse varied across the groups; group D had the highest percentage of sexually active women. In group A only two patients were sexually active, while in group C none. Overall, among those sexually active, 57% presented FSD, revealed by FSFI. However, no differences were found in total FSFI score or in its subdomains based on karyotype or therapy regimen; FSFI scores are reported in Table 8. Of the 15 sexually active patients, all but one reported being in a stable relationship with a partner. Among the 15 non-sexually active patients, 2 reported being in a stable relationship and stated that they did not engage in sexual activity by personal choice. These patients also declared that their partners did not have any sexual dysfunctions. Higher SHBG levels were directly correlated with better scores in the pain domain of FSFI and a less frequently pathological FSFI overall score (*p* = 0.003, *p* = 0.027). No significant differences in FSFI scores, including arousal, lubrication, or other domains, were observed based on the presence or absence of antihypertensive therapies, lipid-lowering drugs, or medications for glucose intolerance and type 2 diabetes (e.g., metformin). Autoeroticism was reported by 7 women with TS, at least occasionally; 5 of these women were sexually active. Among the patients, all reported practicing masturbation at a frequency of 1–2 times per month, except for one patient who reported a frequency of 3–7 times per month. There were no significant differences based on group classification: three patients were from Group B, one from Group D, one from Group C, and one from Group A. The FSFI scores in these women did not differ from those who did not engage in autoeroticism.

**Table 8:** Overall FSFI parameters

FSFI parameters	
FSFI desire	2.9 (2.5, 3.3)
FSFI arousal	4.3 (3.6–5.1)
FSFI lubrication	4.8 [3.6–5.1]
FSFI orgasm	4.1 (3.5, 5.0)
FSFI satisfaction	5.2 [2.3-6.0]
FSFI pain	3.9 (3.2, 4.5)
FSFI total score	26.0 [22.7–27.6]

Values are expressed as means and 95% confidence intervals in parentheses, or as median and interquartile range (IQR) in square brackets

### Body perception

A total of 44.8% of participants exhibited a Global Severity Index (GSI) > 1.2, indicative of a body image disorder **(**Table 9**)** [29, 32]. No differences were observed in the survey outcomes across the groups. Furthermore, BUT-A domain results did not vary with HRT regimen used, previous rhGH treatments, or according to condition of overweight or obesity. The Avoidance domain of BUT-A was correlated with pathological scores on FSFI (*r* = 0.628, *p* = 0.016); whereas lower pathological scores were associated with higher TT levels (*r*= -0.403, *p* = 0.034). The actual height of the patients showed no significant correlation to the BUT-B question addressing height (*r* = − 0.25, *p* = 0.09). Similarly, no significant differences in the distribution of height scores were observed across FSFI pathological categories (*p* = 0.524) or between patients reporting active sexual activity and those who did not (*p* = 0.085).

**Table 9:** BUT parameters in patients with TS grouped by karyotype.

Body image concerns	Group A	Group B	Group C	Group D	Total	*p*
Global severity index	1.0 [0.4–2.3]	1.5 [0.9–2.6]	1.0 [0.7–1.8]	2.0 [0.6–2.3]	1.1 [0.6–2.3]	0.731
Weight phobia	1.8 (0.4–3.2)	1.9 (1.1–2.7)	1.4 (0.1–2.7)	2.3 (1.0-3.8)	1.9 (1.4–2.4)	0.687
Body image concerns	1.0 [0.8-3.0]	2.0 [1.0-2.6]	1.4 [1.0-2.9]	1.8 [1.1–3.5]	1.6 [1.0-3.2]	0.731
Avoidance	0.3 [0.0-1.2]	0.5 [0.0-1.8]	0.4 [0.2-1.0]	0.7 [0.2–1.6]	0.5 [0.2–1.3]	0.888
Compulsive self-monitoring	0.6 [0.2–1.6]	1.2 [0.6–2.4]	0.8 [0.8–1.5]	1.2 [0.4–2.8]	0.8 [0.5–2.1]	0.672
Depersonalization	0.8 [0.0-1.3]	0.7 [0.2–1.5]	0.6 [0.5–0.9]	0.8 [0.0-1.3]	0.7 [0.1–1.2]	0.969
Positive symptom total	2.8 (0.4–5.2)	6.0 (4.8–7.1)	4.5 (0.5–8.5)	5.1 (3.7–6.6)	4.8 (3.9–5.7)	0.058
Positive symptom distress index	0.4 (0.1–0.7)	0.8 (0.6–0.9)	0.6 (0.1-1.0)	0.6 (0.4–0.8)	0.6 (0.5–0.7)	0.058

Values are expressed as means and 95% confidence intervals in parentheses, or as median and interquartile range (IQR) in square brackets.

## Discussion

This study highlights the intricate relationship between sexual function, androgen levels, and body image perception among women with TS, recognizing the impact of significant phenotypic differences associated with distinct karyotype. These results underscore the potential contribution of hormonal imbalances to body image dissatisfaction and sexual dysfunction in patients with TS.

To fulfil this purpose, participants were classified into four groups based on karyotype distinctions, as similarly performed in a Chinese study conducted in 2019 [33].

Frequency of spontaneous menarche varied according with karyotype: all 45, X monosomy required puberty induction, whereas those with some 46, XX cell lines mostly experienced spontaneous menarche. Individuals with mosaic patterns or structural alterations of X chromosome were less likely to require puberty induction than those with mosaic patterns [4]. Consistent with these results, Cameron-Pimblett and colleagues reported a lower primary amenorrhea prevalence in patients with X isochromosome and X ring chromosome compared to those with 45, X karyotypes [4].

In another study, patients were categorised according to the karyotypes into groups 45, X, 45, X/46, XX, corresponding to our groups A and D respectively, and a “miscellaneous” group equivalent to our groups B and C. This study showed a decreasing trend in the onset of ovarian insufficiency from the monosomy group to the mixed group, and further to the group with some 46, XX cell lines [10]. We further demonstrated a greater uterine volume in women with spontaneous menarche compared to those requiring puberty induction, emphasizing the importance of achieving sufficient uterine growth, especially for fertility considerations [1]. However, uterine volume did not reach normal levels in patients undergoing puberty induction and remained below the average size reported in nulliparous, non-hypogonadal women [34].

The feasibility of achieving normalisation of uterine volume varies across studies, with some reporting it as achievable with tailored pubertal induction protocols, whereas others report persistent limitations [35–37]. However, this effect was not observed in this cohort, possibly due to incomplete dosing details and variability in pubertal induction protocols, which limit definitive conclusions. Lastly, no karyotype-based differences in uterine dimensions were found, in agreement with prior research [37].

Hormonal analyses revealed lower levels of cfT, FAI and DHEA-S in Group A compared to group D. These findings are consistent with the known effects of “streak gonads,” where reduced ovarian function leads to minimal androgen production, particularly ∆4 and TT [38]. Notably, within the group that experienced spontaneous menarche, TT, cfT, FAI, and DHEA-S levels were significantly higher. This supports the notion that spontaneous ovarian activity correlates with increased androgen production. However, the reduced levels of DHEA-S, primarily secreted by the adrenal glands, suggest that adrenal androgen production could also be compromised in patients with TS. This phenomenon appears particularly evident in patients with a pure 45,X karyotype, often associated with a more severe clinical phenotype. The greater severity of ovarian dysgenesis in these patients might inherently affect adrenal function, potentially through shared developmental or hormonal pathways. Lower adrenal androgen levels in these patients could also reflect systemic factors, such as metabolic stress, elevated insulin levels, chronic inflammation, or coronary pathologies, all of which have been linked to reduced DHEA-S production in other populations [39–41]. Consequently, patients with more severe karyotypes may exhibit a broader disruption of androgen levels extending beyond the ovaries. These results are consistent with those reported in the recent study conducted by Viuff et al., the most extensive research investigating serum levels of sexual hormones in patients with TS [18]. However, the Danish study included only three patients receiving TD therapy and no subdivision based on karyotype was performed. Some research suggests that exogenous oestrogens in HRT might reduce DHEA-S levels, but the exact mechanism remains unclear [42, 43]. These observations underscore the multifactorial nature of hormonal imbalances in TS, highlighting the need for further studies to elucidate the underlying mechanisms driving androgen reductions and their interaction with karyotype, adrenal function, and systemic factors.

It is well known that oral HRT induces an increase in SHBG, leading to a further decrease in bioavailable androgens [44]. The higher levels of SHBG observed in the oral therapy group confirm existing data in literature and highlight a greater hepatic effect of oral oestrogens than TD formulation [45]. Surprisingly, in contrast to existing literature, reduction of LH levels was greater in TD vs. oral route [46]. This, in our opinion, could be attributable to the poor compliance, especially in the early stages of TD HRT, that may be observed among patients.

To date, this research is one of the few studies that assess sexual function in patients with TS, firstly indicating that about 50% of patients are sexually active, in line with the previous literature [25, 47]. Our findings diverge from other studies on the presence of FSD in sexually active patients [25–27, 47, 48]. For instance, in a study conducted by Ros and colleagues, sexually active patients with TS showed scores comparable to both a control population and patients with hypogonadotropic hypogonadism, except for the arousal domain; however, this study did not provide scores for individual domains or the percentage of patients with a pathological response to the FSFI questionnaire [25]. In the investigation carried out by van den Hoven et al., it was reported that 80% of patients with TS expressed satisfaction with their sexual life, although specific data were not provided. Although four different questionnaires related to sexual function, including FSFI, were administered, none of the results were reported, and the definition of satisfaction remained unspecified [47]. Contrasting with previous literature, suggesting a satisfactory sexual life in patients with TS, our study indicates a high prevalence of FSD, potentially linked to relational and physical challenges. This information emerged not only through the survey responses but was also communicated by the patients themselves during their visits. We believe this may be due to the importance attached to this topic during our medical consultations and an appropriate care setting. In this cohort, seven women, five of whom were sexually active, practised autoeroticism. A previous study that explored the likelihood of masturbation in these patients reported lower rates than in healthy controls or individuals with other disorders of sexual development, but comparable to those observed in individuals with partially virilising XY disorders [49]. However, the percentage identified in another study was higher compared to that emerged from our study, yet it remained lower than the general population [27]. About this issue, a recent Spanish study included an interview conducted among patients with TS, revealing the perception that healthcare professionals were not interested in their sexual activity, relationship with their body, and masturbation, “experienced their sexuality under a cloak of silence” [50].

No differences in FSFI scores were observed between patients practising autoeroticism and others. This could be clarified by other unexamined factors; for example, in a recent survey, women who practised autoeroticism more frequently were also less satisfied with sexual activity with their partner [51]. Conversely in other studies, women practising autoeroticism were found to be more sexually active and more satisfied with sexual activity [52].

Among the participants, higher SHBG levels were associated with improved pain scores and less frequent pathological FSFI responses. These findings suggest that SHBG may serve as a marker of oestrogenic activity and underline the significant role of oestrogens in influencing specific domains of sexual function, particularly pain modulation [53]. However, androgen levels did not show significant correlations with overall FSFI scores.

We found no differences in body image perception outcomes across various chromosomal anomalies, and about 45% of the study sample exhibited a potential disorder in body image perception [29]. This corresponds with observations from the limited available literature, including a 2020 study reporting a comparable prevalence of 37% among patients with TS [27, 28]. The results related to karyotype are partly conflicting compared to those obtained from a similar study conducted on adolescent girls with TS; however, the latter study employing a different questionnaire, the “Multidimensional Body-Self Relations Questionnaire”, demonstrated that patients with a pure karyotype had a poorer perception of body image compared to those with mosaic karyotype. Nevertheless, this observation was not confirmed in the multiple linear regression analysis including age, race, and socio-economic status [28].

Avoidance behaviours, as measured by the BUT-A, were correlated with pathological FSFI scores, highlighting the associations between body image discomfort and sexual distress. This is consistent with evidence from non-TS cohorts, where body image dissatisfaction has been shown to significantly impact sexual well-being [54]. Additionally, lower testosterone levels in this population were inversely correlated with higher pathological BUT-A scores, suggesting that testosterone may play a critical role in modulating body image perception [54]. These results align with a pilot study conducted by Zuckerman-Levin et al., which demonstrated that androgen replacement therapy in women with TS improved aspects of cognitive function, quality of life (QoL), and sexual desire [55]. Collectively, the data suggest that oestrogens, mediated by SHBG, predominantly influence physical aspects like pain, whereas androgens may be more critical for psychological and relational dimensions of sexual health. The interplay between oestrogens and androgens in female sexual health is multifaceted. Oestrogens primarily influence lubrication and pain modulation through central and peripheral mechanisms and androgens are more directly involved in sexual desire and arousal, acting via the dopaminergic reward system ​ [53, 56]. In this cohort, elevated SHBG levels likely reflect increased oestrogenic activity, contributing to improved pain scores. However, the observed reduction in bioavailable androgens could limit benefits related to sexual desire and body image perception. This highlights the distinct yet complementary roles of these hormones, underscoring the need for further studies to explore optimal hormonal strategies in TS patients.

Finally, the role of height in body image was also explored, revealing a non-significant trend suggesting that shorter stature might contribute to a more pathological perception of height. This observation is consistent with studies highlighting the psychological challenges associated with short stature in TS [57, 58]. However, adult height has not been found to significantly influence overall QoL in TS [58]. Moreover, recent evidence suggests that body image challenges in TS are multifactorial, involving not only physical traits like height but also broader psychosocial and relational factors [28].

This study has several limitations that should be acknowledged. The main limitation is represented by the small sample size, although it is crucial to acknowledge that TS is a rare disease. Another major limitation of this study is that androgen levels were not measured using mass spectrometry. Additionally, the duration of HRT was not uniform, and this may have partially influenced the results. Nevertheless, the minimum therapy duration at the time of evaluation was one year. Moreover, the lack of assessment of sexual-related distress represents another limitation of this study. Although tools such as the Female Sexual Distress Scale are available for evaluating this parameter, no officially validated Italian version currently exists, which influenced our decision not to include it. An additional limitation is that, although orgasmic function was evaluated in this study using the FSFI, we acknowledge the absence of a psychometric tool specifically validated to measure orgasmic intensity, such as the Orgasmometer-F [59]. Finally, we encountered challenges in obtaining detailed information about pubertal development, dosing and time used for pubertal induction and our assessment was limited to relying exclusively on the occurrence or absence of spontaneous menarche.

## Conclusions

To the best of our knowledge, this is the first study that concurrently investigates sexual function in relation to androgen levels and body image perception, in patients with TS, according to the karyotype. We observed phenotypic crucial differences based on karyotype within the cohort underscoring the importance of tailoring treatments in rare diseases management. Furthermore, individuals with TS may experience low satisfaction with their body image and most sexually active patients present FSD.

The observation of decreased androgen levels in patients with TS receiving oral therapy suggests that a potential shift towards TD therapy could be beneficial. Although this route of administration might be deemed less convenient by some patients and is yet to be strongly substantiated by extensive research, it is supported by solid physiological premises. The findings also highlight the importance of an oestrogen-rich environment in supporting sexual health, with androgens playing a complementary but often overlooked role.

This study adds new insights in TS research, particularly in less explored areas such as sexuality and body image perception, underlying the need for more comprehensive and multidisciplinary care aimed to improve these patients’ QoL.
